# Induction of neuroplasticity and recovery in post-stroke aphasia by non-invasive brain stimulation

**DOI:** 10.3389/fnhum.2013.00888

**Published:** 2013-12-24

**Authors:** Priyanka P. Shah, Jerzy P. Szaflarski, Jane Allendorfer, Roy H. Hamilton

**Affiliations:** ^1^Department of Neurology, University of PennsylvaniaPhiladelphia, PA, USA; ^2^Laboratory for Cognition and Neural Stimulation, Center for Cognitive Neuroscience, University of PennsylvaniaPhiladelphia, PA, USA; ^3^Department of Neurology, University of Alabama at BirminghamBirmingham, AL, USA

**Keywords:** TMS, rTMS, fMRI, tDCS, rehabilitation, aphasia

## Abstract

Stroke victims tend to prioritize speaking, writing, and walking as the three most important rehabilitation goals. Of note is that two of these goals involve communication. This underscores the significance of developing successful approaches to aphasia treatment for the several hundred thousand new aphasia patients each year and over 1 million stroke survivors with chronic aphasia in the U.S. alone. After several years of growth as a research tool, non-invasive brain stimulation (NBS) is gradually entering the arena of clinical aphasiology. In this review, we first examine the current state of knowledge of post-stroke language recovery including the contributions from the dominant and non-dominant hemispheres. Next, we briefly discuss the methods and the physiologic basis of the use of inhibitory and excitatory repetitive transcranial magnetic stimulation (rTMS) and transcranial direct current stimulation (tDCS) as research tools in patients who experience post-stroke aphasia. Finally, we provide a critical review of the most influential evidence behind the potential use of these two brain stimulation methods as clinical rehabilitative tools.

## Introduction

Aphasia, defined as an impaired ability to communicate, is one of the most feared symptoms of stroke. About 21–38% of acute stroke survivors suffer from aphasia (Berthier, 2005), a devastating neurological condition affecting a person's ability to communicate and, thus, reintegrate into the society. It is a consequence of damage in a widely distributed and complex language network involving the fronto-temporal areas in the dominant hemisphere (typically left). Aphasia usually impacts all areas of communication including language formulation and comprehension as well as the ability to read and write. These deficits are attributed to damage in higher cognitive areas involved in language processing rather than to areas involved in motor control of the articulatory structures (Allendorfer et al., 2012a), although aphasia and disorders of speech articulation often coincide.

The first 2 to 3 months after stroke are crucial for spontaneous neuroplasticity, which refers to the natural course of neurophysiological repair and cortical reorganization of language functions (Robertson and Fitzpatrick, 2008). During this period, restoration of some language functions is common and usually fairly rapid (Lazar et al., 2008). However, the slope of spontaneous recovery tends to level off within the first year of stroke (Pedersen et al., 1995; Berthier, 2005), resulting in chronic impairments in language processing in many patients.

Despite availability of pharmacological treatments and professionally-administered speech-language therapy (SLT), new strategies e.g., adjuvant therapies, are required to boost recovery, especially in the chronic stages of stroke. While SLT is the most commonly employed treatment of aphasia, its therapeutic effects are quite variable and are generally modest (Berthier, 2005; Brady and Enderby, 2010). Recently, non-invasive brain stimulation (NBS) techniques, including repetitive transcranial magnetic stimulation (rTMS) and transcranial direct current stimulation (tDCS) have shown promise as potential approaches for enhancing aphasia recovery. A number of research studies employing these techniques, especially repetitive rTMS, have reported lasting improvement in specific language functions in patients with chronic post-stroke aphasia. In addition to behavioral improvement, evidence of induced neuroplasticity has further validated the efficacy of these interventions. However, application of therapeutic NBS within few days after stroke i.e., in sub-acute and acute phase, is still in its infancy.

In this article, we will explore the neuroplastic processes that underlie spontaneous recovery in patients with aphasia, and present the methods and discuss the physiologic basis of NBS techniques. Next, we will discuss recently published and influential work in which NBS has been used to enhance recovery from post-stroke aphasia. Lastly, we will review studies that investigate the effect that NBS has on neuroplasticity in patients with aphasia; specifically, we will examine studies that address the functional neuroimaging and electrophysiologic correlates of neuroplastic changes after brain stimulation.

## Neuroplasticity in spontaneous recovery of post-stroke aphasia

Converging evidence indicates that recovery in post-stroke aphasia is supported by compensatory changes in the representation of language functions, either involving recruitment of areas surrounding lesions in the language-dominant left hemisphere, or altered activity of intact homotopic language areas in the non-dominant right hemisphere, or both (Hamilton et al., 2011). Recruitment of previously inactive pathways, appears to apply to motor rather than language recovery and will not be discussed here (Lee and Vandonkelaar, 1995). Recently, modern investigative techniques such as functional magnetic resonance imaging (fMRI), positron emission tomography (PET), electroencephalogram-based event related potentials (EEG-ERP), and diffusion tensor imaging (DTI) have been applied to understand neuroplasticity in the context of spontaneous language recovery after stroke [for details of the application of various neuroimaging techniques to the evaluation of post-stroke recovery see recent review by Eliassen and colleagues (2008)]. In this section, we will discuss the proposed models of neuroplasticity in aphasia recovery with supporting evidence from neuroimaging investigations that capture changes in brain function as a measure of neuroplasticity after stroke.

Based on assembled evidence from prior studies, three models of neuroplasticity underlying aphasia recovery in adults were outlined by Hamilton et al. (2011). These are: (1) recruitment of residual perilesional language areas in the language-dominant left hemisphere (Ohyama et al., 1996; Karbe et al., 1998a,b; Warburton et al., 1999; Cornelissen et al., 2003), (2) compensatory recruitment of homotopic language areas in the non-dominant right hemisphere (Musso et al., 1999; Thulborn et al., 1999; Tillema et al., 2008), and (3) inefficient recruitment of sites in the non-dominant right hemisphere, which hinders rather than aids recovery (Turkeltaub et al., 2011). In addition, increased involvement of right homotopic language areas due to release from transcallosal inhibition may also negatively affect spontaneous neuroplasticity. By this account, interhemispheric inhibitory connections that normally modulate and effectively suppress right hemispheric activity are disturbed due to damage in the left hemisphere, enabling areas in the contralesional right hemisphere to become increasingly involved via disinhibition. It has been proposed that increased involvement of right hemispheric regions in post-stroke language production in adults may exert an increased inhibitory influence on perilesional areas in the left hemisphere, interfering with ability of these regions to contribute to language recovery (Belin et al., 1996; Rosen et al., 2000; Naeser et al., 2004). This last model provided the rationale for a number of studies in which suppression of right hemispheric activity or stimulation of the left hemispheric peri-stroke areas with NBS has been employed in order to enhance language performance in patients with aphasia (Naeser et al., 2005; Kang et al., 2011; Szaflarski et al., 2011a,b; Marangolo et al., 2013). As outlined below, most of these studies target specific sites in the right hemisphere.

Evidence regarding the role of the right hemisphere in language recovery is mixed; while some studies suggest that recruitment of right homotopic areas may be beneficial or compensatory in nature (Thulborn et al., 1999; Tillema et al., 2008), other studies show that activation of these right hemisphere regions during language performance may indicate a maladaptive strategy of recovery (Winhuisen et al., 2005; Thiel et al., 2006). For instance, Winhuisen et al. (2005) found that right-hemispheric involvement may only be partially compensatory in sub-acute patients with aphasia. They applied inhibitory rTMS (4 Hz, 10 s trains) to the right and left inferior frontal gyrus (IFG) in 11 patients, where the exact loci of IFG stimulation were based on maximum functional activation on PET during a language task. They showed that individual patients' response to rTMS with left vs. right IFG varied: 8 patients showed increased naming reaction time (RT) with left IFG while 4 patients showed increased RT with right IFG stimulation. Interestingly, the group that showed increased latency after left IFG stimulation performed significantly better on a verbal fluency task than the group that responded to right IFG stimulation. Based on this finding, the authors suggested that patients with residual language function in the left hemisphere, functionally defined by an inhibitory response to rTMS, performed better on the language task than those with right hemispheric involvement. Their findings further suggested that recovery of function in the dominant left hemisphere may be essential for optimal language reacquisition after aphasia, while right hemisphere recruitment may only be partially compensatory or may be maladaptive in some cases.

The maladaptive role of at least one specific site in the right homotopic language areas was recently suggested by Turkeltaub et al. (2011). The authors employed Activation Likelihood Estimation (ALE) meta-analysis of functional neuroimaging studies involving language tasks in 105 patients with chronic aphasia and 129 control subjects. While control subjects showed functional activation patterns predominantly in left perisylvian language areas, patients with aphasia consistently involved spared left hemisphere areas as well as right hemispheric homotopic language areas. While recruitment of some right hemispheric homotopic areas appeared to be beneficial with respect to language performance, activation of the right pars triangularis (PTr; Brodmann areas 45 in the IFG) was found to be inefficient, perhaps even deleterious, with respect to language performance. This finding corroborates the observed therapeutic effects of inhibitory rTMS of right PTr on several language functions, which in effect may act due to suppression of “noisy” or maladaptive activation of the right PTr (Naeser et al., 2005; Barwood et al., 2011a, 2013; Weiduschat et al., 2011; Kindler et al., 2012; Medina et al., 2012; Thiel et al., 2013).

Evidence further suggests that the neuroplastic mechanisms that underlie spontaneous recovery vary greatly among patients with aphasia. Prior research suggests that language recovery in adults depends on several clinical factors, such as the extent and location of lesions (Heiss and Thiel, 2006). With small lesions that spare some areas of eloquent cortex, recovery may rely up on recruitment of residual language areas along with increased perilesional activity. By contrast, in the case of large lesions that engulf primary language regions, recovery may rely on recruitment of homotopic non-dominant language areas (Rosen et al., 2000; Heiss and Thiel, 2006).

Data also indicate that at more than 1 year after aphasia-producing stroke the cortical participation in language production remains relatively stable in absence of intervention. For example, a study utilizing three different fMRI tasks (verb generation, semantic decision, and picture-word matching) assessed language recovery mechanisms in a group of chronic aphasic patients by comparing blood-oxygen-level-dependent (BOLD) signal changes over a period of 10 weeks (Eaton et al., 2008; Szaflarski et al., 2011a). Four chronic patients and an equal number of age-matched healthy controls underwent 5 fMRI sessions (2 runs of each fMRI task per session) over a course of 10 weeks. As expected, patients with aphasia performed worse than the controls on these tasks. In addition, these differences in language performance were associated with differences in cortical activity between the two groups. While control subjects exhibited overall typical bilateral, left greater than right activation in the frontal and temporal language areas as well as symmetric retrosplenial and posterior cingulate areas, the stroke patients exhibited increased and consistent activation in perilesional areas with minimal activation in the contralesional right hemisphere. The authors concluded that among patients with chronic aphasia activation of the left hemispheric language regions and deactivation of the right homologs suggests cortical reorganization after stroke. They further posited that activity in the perilesional areas, rather than in the non-dominant homologs may be the more critical mechanism for language recovery.

More direct evidence of hemispheric changes in brain activity over time is provided in a study by Saur et al. (2006). In a group of 14 patients with post-stroke aphasia, Saur et al. examined the neural correlates of language recovery. They evaluated language task-related fMRI activation patterns in these patients at acute (average of 1.8 days after stroke), sub-acute (average of 12.1 days after stroke), and chronic (average of 321 days after stroke) stroke stages (Saur et al., 2006). In the acute phase, they observed little or no perilesional activation of undamaged areas in the left hemisphere. Whereas in the sub-acute phase, a large increase of activation in a bilateral language network was observed with a peak in the right Broca's homolog (Heiss et al., 1999; Thulborn et al., 1999; Winhuisen et al., 2005) and right supplementary motor area; these increases were strongly associated with improved performance on language tasks. Further, since the authors observed that language improvement in the chronic phase was associated with a redistribution of activation toward the dominant left-hemispheric language areas, recruitment of right hemisphere language homologs may suggest their beneficial role for the early but not the late language recovery. Based on this finding, the authors suggested that the involvement of the right hemispheric areas in recovery may be transient before more favorable perilesional recruitment takes place. However, the exact role of right language homologs during the sub-acute stage of recovery is still unclear. These findings are potentially consistent with the notion that recruitment of right hemispheric areas may only be partially compensatory, and that optimal neuroplastic changes eventually involve recruitment of perilesional areas.

This hypothesis was recently addressed directly by Szaflarski et al. (2013). While Saur et al. (2006) focused on a group of patients who eventually experienced recovery in their language functions at the chronic stage, Szaflarski et al. evaluated the neural correlates of good vs. poor recovery in a group of 27 chronic stroke patients (Saur et al., 2006; Szaflarski et al., 2013). Similar to the findings in Saur et al. (2006), normalization of language functions at least 1 year after stroke was associated with typical fMRI activation patterns i.e., fMRI activity with left hemisphere distribution when compared to healthy controls. However, the reorganization of the language function in the non-recovered group was characterized by activation patterns in the right hemispheric areas. Specifically, increased activity in the left superior frontal and parietal areas and bilateral cerebellum was observed in the recovered vs. the non-recovered group. In addition, a decrease in activation was found in the right superior temporal areas in the recovered vs. non-recovered group. Language performance and the level of hemispheric activation were also associated, in that increase in activation in the right areas was associated with poor trajectory of performance, while increase in activation in the left areas was associated with improved performance. Lesion size also affected language performance consistent with the theory of regional hierarchy (Heiss and Thiel, 2006). Overall, the authors posited that the recruitment of right areas in the poor recovery group may be an indication of a maladaptive or an inefficient pattern of language recovery.

In short, the balance of evidence leads us to conclude that sparing of language areas in the lesioned left hemisphere and/or cortical reorganization of brain activity during recovery to the left hemispheric perilesional areas may be the optimal mechanism of neuroplastic changes with respect to language outcomes. However, the importance of right hemispheric homologs to the process of recovery is not clear. Although the evidence from neuroimaging studies suggests a negative association between language recovery and right hemispheric activation, we posit that involvement of some of the right hemispheric areas may not be deleterious to language recovery and that a specific “noisy” or inefficient site may hinder the downstream recruitment of perilesional and residual language areas, and, therefore, adversely impact recovery (Turkeltaub et al., 2011).

## Neurorehabilitation with non-invasive brain stimulation

The field of neurorehabilitation broadly aims to develop therapies that: (1) derive from an understanding of the mechanisms of healthy brain function and neurological dysfunction after a brain injury, and (2) improve not only behavioral or cognitive performance but also the function of neural systems, which translates into favorable outcomes on everyday quality of life (Robertson and Fitzpatrick, 2008). In light of these goals, NBS techniques provide a unique opportunity for neurorehabilitation after stroke. In the recent years, investigation of NBS techniques to promote stroke recovery has grown immensely and is continuously supported by the advent of new technologies. The application of NBS specifically to post-stroke aphasia rehabilitation leverages current understanding of the models of spontaneous language plasticity discussed above with various neuroimaging techniques such as fMRI (Szaflarski et al., 2011a,b) or EEG-ERP (Barwood et al., 2011b,c) to provide further evidence of stimulation-induced neuroplasticity. Also, insofar as improving function in everyday life is of paramount concern to neurorehabilitation, changes in functional communication outcomes after therapeutic NBS, have been assessed in at least 2 studies (Szaflarski et al., 2011b; Marangolo et al., 2013), where patients with aphasia tended to report improved ability in communication after tDCS and rTMS.

In this section, we will introduce basic principles of TMS and tDCS and summarize the current literature describing the therapeutic effects of these two technologies in stroke patients with aphasia (See Table 1). In a subsequent section, we will summarize the accounts of rTMS-induced neuroplasticity.

**Table 1:** **Summary of non-invasive brain stimulation intervention studies for post-stroke aphasia**.

**Study**	***N***	**Stroke onset**	**Methods**	**FU**	**Site**	**Tests**	**Findings—significant improvement in:**
**rTMS: CASE REPORTS/SERIES**							
Naeser et al., 2010	1	Chronic	1 Hz rTMS 90% RMT 10 days 20 min/day Optimal site finding CPAP	3, 6, 2.4 years	Right PTr (5 sites: Motor mouth area, and 4 subregions within Broca's area)	Picture naming, BDAE, BNT	Phrase length, auditory comprehension and BNT at 3 and 6 months post-TMS
Cotelli et al., 2011	3	Chronic	20 Hz rTMS 90% RMT 10 or 20 days 25 min/day 25 min of SLT	About 1, 3, 6, 11	Left dlPFC	AAT, BADA, picture naming, verbal fluency and reasoning	Picture naming accuracy; persistent benefit present 48 weeks after treatment
Hamilton et al., 2010	1	Chronic	1 Hz rTMS 90% RMT 10 days 20 min/day Optimal site finding	2, 6, 10	Right PTr (sites: POp, dpPTr, vpPTr, aPTr, PO, Motor mouth area)	WAB, BDAE-Cookie theft, picture naming	Naming and spontaneous speech; improvement in picture description sustained at 2, 6 and 10 months
Barwood et al., 2012	7	Chronic	1 Hz rTMS 90% RMT 10 days 20 min/day	2, 8	Right PTr	BNT, BDAE, picture naming	Naming accuracy and latency, generalized speech output, and auditory speech comprehension; effects sustained up to 8 months
Martin et al., 2009	2	Chronic	1 Hz rTMS 90% RMT 10 days 20 min/day Optimal site finding fMRI: changes in activation patterns	2, 6, 16, 43	Right PTr (4 sites: POp, aPTr, pPTr, Motor mouth area)	BNT, BDAE—Cookie theft, picture naming	Picture naming and phrase length in one patient (responder; best response site right pPTr); No improvement in the other patient (non-responder; best response site right aPTR)
**rTMS: GROUP STUDIES**							
Naeser et al., 2005	4	Chronic	1 Hz rTMS 90% RMT 10 days 20 min/day	2, 8	Right PTr	BNT, BDAE, picture naming	Picture naming at both 2 and 8 months in 3 patients
Kakuda et al., 2011	4	Chronic	10 min of 6 Hz followed by 20 min of 1 Hz rTMS 90% RMT 11 days 2 sessions/day (except on 1st and last day) 60 min of SLT	−	Right IFG (F8)^*^	SLTA, J-WAB	(greatest) repetition and naming; 4 patients showed improvement in different categories including naming, repetition, writing, auditory and visual comprehension and speech; none showed deterioration
Barwood et al., 2011a	6 real 6 sham	Chronic	1 Hz rTMS 90% RMT 10 days 20 min/day	2	Right PTr	BNT, BDAE, picture naming	Naming, aspects of expressive language and auditory comprehension
Medina et al., 2012	5 real 5 sham (crossed-over to real after 2 months)	Chronic	1 Hz rTMS 90% RMT 10 days 20 min/day Cross-over Optimal site finding	2	Right IFG (sites: POp, dpPTr, vpPTr, aPTr, PO, Motor mouth area)	BDAE, BNT, narrative speech production	Fluency at 2 months after rTMS, specifically in discourse productivity; no benefit in sentence complexity, grammatical accuracy or lexical selection; for 9/10 patients, the optimal site was right PTr
Kindler et al., 2012	18	Sub-acute and Chronic	cTBS (3 pulses at 30 Hz) 2 days—Sham/real Cross-over 44 s/day	−	Right PTr	Timed picture naming, alertness task	Naming and reaction time after TBS vs. sham; no differences observed in arousal; patients in sub-acute phase were best responders
Waldowski et al., 2012	13 real 13 sham	Sub-acute	1 Hz rTMS 90% RMT 15 days 30 min/day 45 min SLT	3.5	Right PTr and right POp	ASRS, BDAE, picture naming^7^	Aphasia severity (ASRS) in real group compared to the sham 15-weeks after treatment; naming accuracy did not differ between groups but reaction time was slightly faster in the real group after treatment; real subgroup with lesions involving frontal area showed slower reaction times
Barwood et al., 2013	6 real 6 sham		1 Hz rTMS 90% RMT 10 days 20 min/day	2, 8, 12	Right PTr		Naming, expressive language and auditory comprehension up to 12 months in the real group compared to sham
Barwood et al., 2011b	6 real 6 sham	Chronic	1 Hz rTMS 90% RMT 10 days 20 min/day N400 ERP	2	Right PTr	BNT, BDAE, picture naming, SJT	(Differences in) mean and peak amplitudes and area under the curve measures of N400 ERP component between real and sham group at 2 months
Weiduschat et al., 2011	6 real 4 ctrls	Sub-acute	1 Hz rTMS 90% RMT 8–10 days 20 min/day PET SLT	−	Right PTr or Vertex	AAT	AAT; activation shift toward right hemisphere in control group, absent in intervention group; laterality shift and clinical improvement were not related
Szaflarski et al., 2011b	8	Chronic	fMRI-guided iTBS (3 pulses at 50 Hz) 10 days 200 s/day 80% AMT LI	−	Left PTr	BNT, SFT, COWAT, PPVT, CAL, BDAE CompId	SFT; activation shifts to the affected left hemisphere; self-reports of improved communicative ability (tendency)
Allendorfer et al., 2012b	8	Chronic	fMRI-guided iTBS (3 pulses at 50 Hz) 10 days 200 s/day 80% AMT DTI—FA	−	Left PTr	BNT, SFT, COWAT, PPVT, CAL, BDAE CompId	SFT; higher DTI-FA values in the left fronto-temporo-parietal areas
Abo et al., 2012	24	Chronic	fMRI (right or left) and aphasia type (STG or IFG)-guided 1 Hz rTMS 90% RMT 10 days 40 min/day 60 min of SLT	−	Fluent: Right STG (CP6^*^; *n* = 5), Left STG (CP5; *n* = 5); Non-fluent: Right IFG (F8; *n* = 11)Left IFG (F7; *n* = 3)	SLTA, J-WAB	Auditory and reading comprehension, and repetition in non-fluent aphasia patients; improvement in spontaneous speech in fluent aphasia patients
Thiel et al., 2013	13 real 11 sham	Sub-acute	1 Hz rTMS 90% RMT 10 days 20 min/day PET 45 min of SLT	−	Right PTr or Vertex	AAT	Global AAT and naming subtests; larger activation index in the left hemisphere in rTMS group post-treatment compared to the sham group
**tDCS: GROUP STUDIES**							
Monti et al., 2008	4 a-tDCS 4 c-tDCS	Chronic	a-tDCS and c-tDCS 2 mA 10 min Reference on right shoulder Cross-over	−	Left fronto-temporal (crossing point between T3-Fz and F7-Cz)^*^	Picture naming task	Picture naming accuracy after c-tDCS whereas a-tDCS and sham induced no changes
Baker et al., 2010	10	Chronic	a-tDCS and sham 1 mA 5 days/condition 20 min/day Reference on right shoulder Cross-over Online anomia treatment^4^	−	fMRI-guided left frontal areas	Picture naming task, WAB-R, ABA-2	Naming accuracy after a-tDCS compared to sham; effects persisted at least 1-week post-treatment
Fiori et al., 2011	3	Chronic	a-tDCS and sham 1 mA 20 min/day 5 days/condition Online language training	−	Left Wernicke's	Picture naming task, BADA	Picture naming accuracy with a-tDCS and sham; shorter naming latencies during a-tDCS compared to sham. Accuracy and RT were better at 1 and 3 weeks after a-tDCS
Fridriksson et al., 2011	8	Chronic with posterior lesions	a-tDCS and sham 1 mA 5 days/condition 20 min/day Reference on right forehead Cross-over Online anomia treatment^4^	−	fMRI-guided (left) perilesional areas	Picture naming task	(Reduction in) reaction times during naming after a-tDCS compared to sham; effects persisted at least 3-weeks
Floel et al., 2011	12	Chronic	a-tDCS, c-tDCS, and sham 1 mA 20 min/day 3 days/condition Cross-over Reference in contralateral supraorbital Online anomia training	−	Right temporo-parietal	AAT; picture naming	Picture naming accuracy after all stimulation conditions observed with anomia training; sustained benefits were found 2-weeks after a-tDCS as compared to sham and c-tDCS
You et al., 2011	7 a-tDCS 7 c-tDCS 7 sham	Sub-acute	a-tDCS on left or c-tDCS on right or sham 2 mA 10 days 30 min Reference on contralateral supraorbital Online SLT	−	Left and Right Wernicke's (STG)	K-WAB	Auditory verbal comprehension after right c-tDCS compared to left a-tDCS and sham; overall improvement in AQ and spontaneous speech also observed across groups
Kang et al., 2011	10	Chronic	c-tDCS and sham 2 mA 20 min 5 days/condition Cross-over Reference on left supraorbital Online word-retrieval training	−	Right Broca's homolog	Korean-BNT	Naming accuracy at 1 h after c-tDCS but no changes in sham tDCS
Marangolo et al., 2013	12	Chronic	a-tDCS and sham 1 mA 20 min 10 day/condition Reference on right frontopolar Cross-over Online conversational therapy	1	Left Broca's (F4)^*^ and Wernicke's (CP5)^*^	BADA, token test, ecological measure, attention and memory tests	Informative speech–increase in content-units, verb and sentence production– after a-tDCS on left Broca's area; effects sustained up to 3 months

CPAP, Continuous Positive Airway Pressure; AAT, Aachen Aphasia Test; ABA-2, Apraxia Battery for Adults—Second Edition; AMT, Active Motor Threshold; ASRS, Aphasia Severity Rating Scale; a-tDCS, anodal transcranial direct current stimulation; BADA, Battery for the Analysis of Aphasic Disorders; BDAE, Boston Diagnostic Aphasia Examination; BNT, Boston Naming Test; CAL, Communicative Abilities Log; CompId, Complex Ideation subtest; COWAT, Controlled Oral Word Association Test; cTBS, Continuous Theta Burst Stimulation (inhibitory rTMS protocol); c-tDCS, cathodal transcranial direct current stimulation; ctrl, control; dlPFC, Dorsolateral prefrontal cortex; DTI-FA, Diffusion Tensor Imaging—Fractional Anisotropy; EEG, Electroencephalogram; FU, Follow-up in months after treatment; IFG, Inferior frontal gyrus; iTBS, intermittent theta burst stimulation; J-WAB, Japanese-Western Aphasia Battery; K-WAB, Korean-Western Aphasia Battery; LI, Lateralization index; mA, milliamperes (unit of current); PET, Positron emission tomography; PPVT, Peabody Picture Vocabulary Test; PTr, Pars triangularis (Anterior portion of Broca's area); RMT, Resting Motor Threshold; SFT, Semantic Fluency Test; SJT, Word, picture semantic judgment task; SLT, Speech language therapy; SLTA, Standard Language Test of Aphasia; STG, Superior temporal gyrus; WAB-R, Western Aphasia Battery—Revised.

* EEG International 10–20 system.

### Repetitive transcranial magnetic stimulation (rTMS)

TMS is a focal NBS method, which employs the principle of electromagnetic induction. A TMS stimulator unit consists of capacitors that store large electrical charges, which is connected to a casing with coil of copper wires. For TMS delivery, this coil is held tangentially to the scalp. When the stored charge is discharged to the coil, a brief and time-varying magnetic field is produced at the scalp. This magnetic field penetrates through the skull, and depending on stimulation intensity, coil shape, and coil orientation, an electrical current is generated in the cortical neurons near the coil. This current is sufficient to depolarize neuronal membranes and generate action potentials. TMS can be delivered either via single pulses or repetitively at a set number of pulses per second (repetitive TMS or rTMS). Typically, low-frequency rTMS (<5 Hz) is characterized by decreased cortical excitability, whereas high-frequency rTMS (≥5 Hz) is characterized by enhanced excitability (Pascual-Leone et al., 1998; Fitzgerald et al., 2006). Recently, a new rTMS protocol, theta burst stimulation (TBS), was introduced which can produce longer-lasting and more stable changes in cortical excitability compared to standard rTMS (Huang et al., 2005). While standard rTMS consists of single pulses of stimulation delivered repeatedly over a unit of time, TBS consists of 3 pulses delivered very rapidly (at 50 Hz) every 200 ms, which can either be interrupted every few seconds [intermittent TBS (iTBS)] or can be uninterrupted (cTBS). ITBS typically increases cortical excitability, while cTBS decreases cortical excitability, and such changes in excitability over the motor cortex have shown to last for about an hour with more intense TBS methods (Huang et al., 2005).

**Figure 1:**
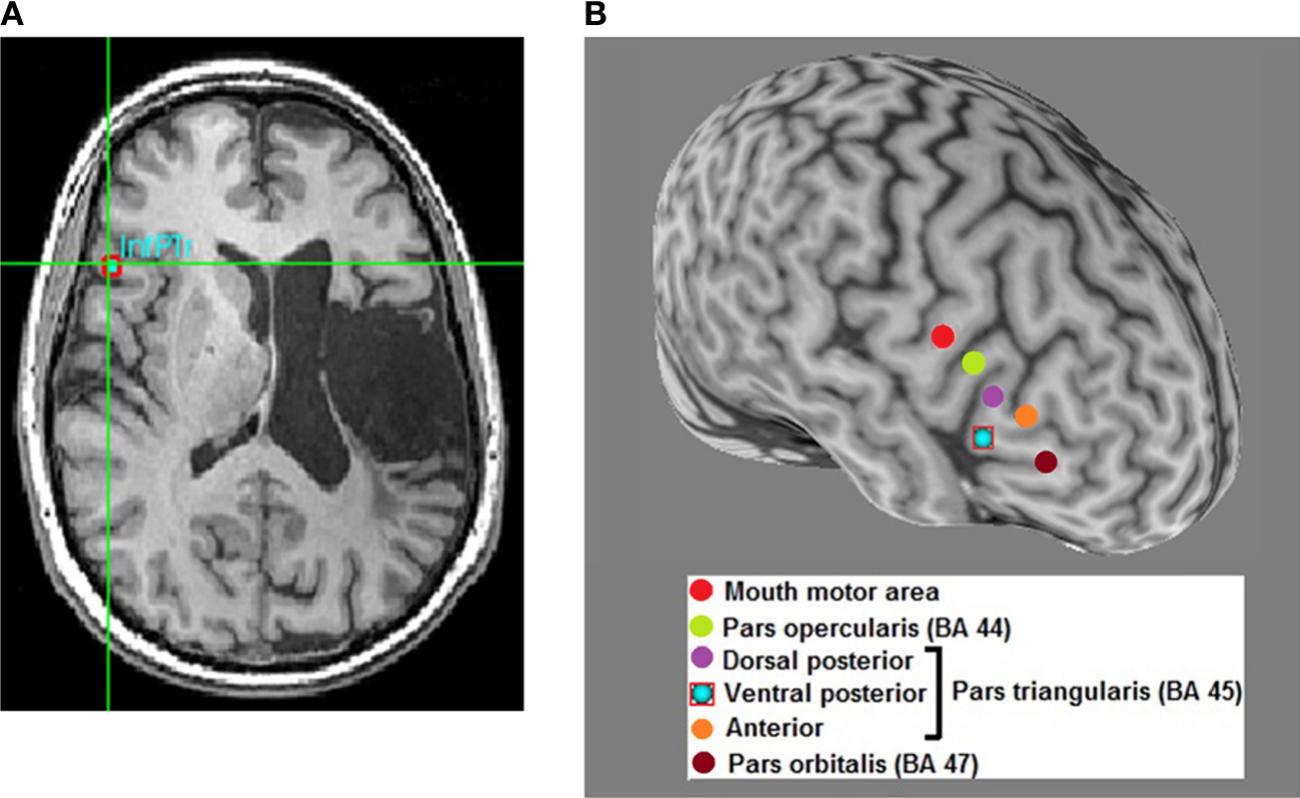
**Optimal site-finding among right hemispheric homolog areas and rTMS in a left hemisphere stroke patient with aphasia. (A)** Among several right hemispheric sites, an optimal site is identified on the subject's high-resolution anatomical scan (red square); optimal site is the one that exhibits better transient language improvement compared to other sites. Most patients respond optimally to the right inferior pars triangularis (InfPTr) site. **(B)** A 3-dimensional reconstruction of the subject's high resolution anatomical scan with the 6 sites-of-interest highlighted in different colors in the right hemisphere. Optimal site for this patient is the ventral posterior (inferior) pars triangularis (PTr).

**Figure 2:**
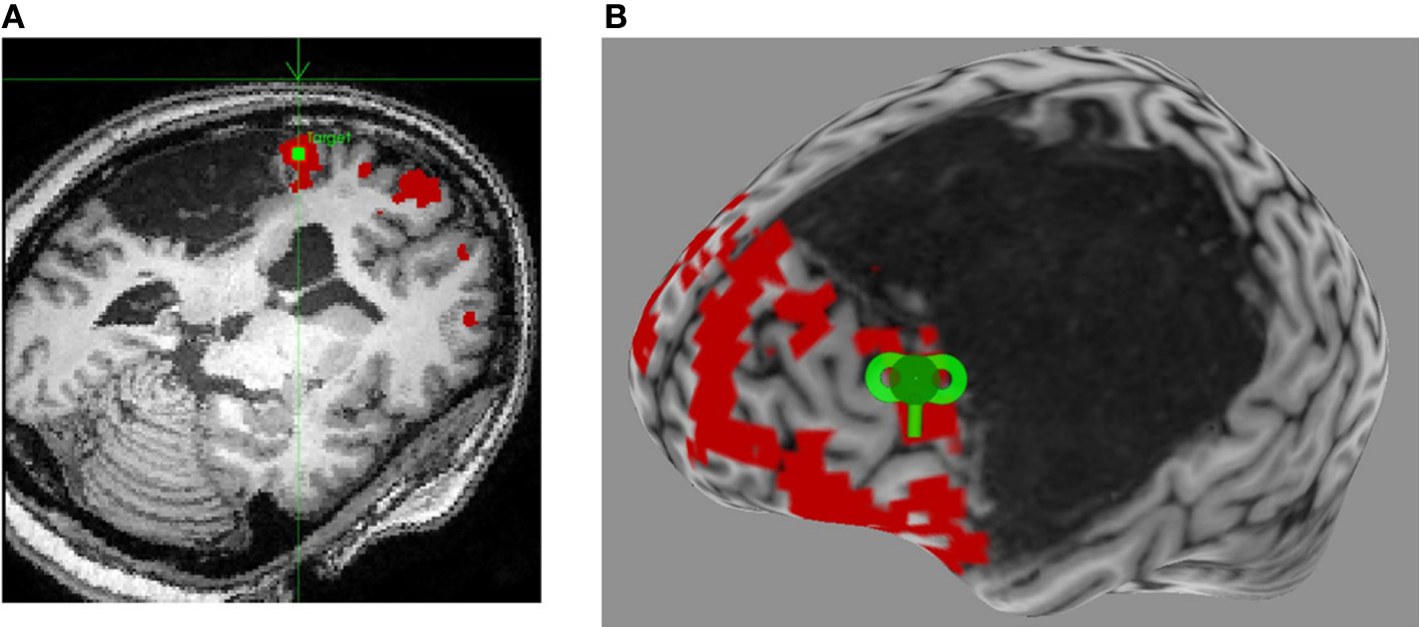
**Neuronavigated rTMS in a left hemisphere stroke patient with aphasia. (A)** Language fMRI activation in left perilesional frontal area is identified on the subject's high-resolution anatomical scan as the stimulation target (green square), and a trajectory is set for optimal stimulation (green arrow). **(B)** A 3-dimensional reconstruction of the subject's high-resolution anatomical scan allows for visualization of the optimal coil placement (green coil) for iTBS.

A review of recent and influential rTMS studies in post-stroke aphasia revealed that most intervention studies administered low-frequency inhibitory rTMS (1–4 Hz) for 20–40 min a day over 10–15 days, on sites in the right hemisphere that were homotopic to left hemisphere sites in the fronto-temporal language network (Broca's or Wernicke's; Figure 1) (Naeser et al., 2005; Barwood et al., 2011a,b,c; Kakuda et al., 2011; Weiduschat et al., 2011; Abo et al., 2012; Kindler et al., 2012; Medina et al., 2012; Waldowski et al., 2012; Barwood et al., 2013; Thiel et al., 2013). Thus, far only 1 group, Szaflarski et al. (2011b), administered iTBS, an excitatory TMS protocol, to increase the cortical excitability in perilesional left-hemispheric language areas (Figure 2) (Szaflarski et al., 2011b). In addition, some studies combined therapeutic rTMS with 45–60 min of speech and language therapy (Kakuda et al., 2011; Abo et al., 2012; Thiel et al., 2013).

Outcome measures, methods of finding the appropriate stimulation site, inclusion of patients by disease duration, and number of long-term follow-up evaluations after rTMS vary considerably between studies. Improvement on subtests of clinical aphasia diagnostic or severity scales [Boston Diagnostic Aphasia Examination (BDAE), Aachen Aphasia Test (AAT), or Western Aphasia Battery (WAB)], Boston Naming Test (BNT), and accuracy and RT in picture naming tasks (Snodgrass and Vanderwart, 1980; Bates et al., 2003) are the most commonly used outcome measures. Most of these studies demonstrated improvement after rTMS in one or more outcome measures. For example, improved picture naming accuracy and RT, auditory comprehension, verbal fluency and repetition have all been observed after daily sessions of low-frequency rTMS (Table 1). In addition, improvement in global scales of aphasia severity was also reported in some studies. These findings clearly suggest the beneficial role of rTMS in improving some language functions in patients with aphasia.

Although right PTr (BA 45), a site in the IFG, was most frequently stimulated, some studies adopted a site-finding protocol (Figure 1) either among several pre-defined right hemispheric sites in individual patients (Martin et al., 2009; Hamilton et al., 2010; Naeser et al., 2011; Medina et al., 2012), or used activation patterns in fMRI (Szaflarski et al., 2011b; Abo et al., 2012; Allendorfer et al., 2012b) to find the most optimal site (Figure 2). For example, Medina et al. (2012) adopted a site-finding protocol similar to Hamilton et al. (2010) and Naeser et al. (2011). They carried out 6 separate rTMS sessions [600 pulses of 1 Hz at 90% resting motor threshold (RMT)] before the daily treatment sessions, where 6 different sites in right IFG were stimulated. Sites included the mouth area in the motor cortex, pars opercularis (POp; BA 44), three separate sites on PTr (dorsal posterior, ventral posterior and anterior PTr), and the pars orbitalis (BA 47). Optimal site for stimulation was determined by evaluating improvement in picture naming accuracy after each stimulation session. A site with the greatest increase in naming accuracy was considered optimal, and patients were stimulated for 10 daily rTMS sessions at this site (1200 pulses of 1 Hz at 90% RMT). Right PTr was the optimal site for 9 of 10 patients, while for 1 patient, right pars orbitalis was the optimal site. After the treatment sessions, the patients that received rTMS improved in several measures of fluency, while patients in the sham group did not improve on any language measures; the beneficial effects persisted for at least 2 months after the treatment ended. (Szaflarski et al., 2011b) adopted an fMRI-based activation approach to finding optimal stimulation sites. Perilesional stimulation targets were identified as regions that exhibited increased activation on fMRI during a semantic language task. Subsequently, iTBS was delivered to each patient's target site in 10 daily sessions lasting 200 s (3 pulses at 50 Hz given every 200 ms in 2 s trains for a total of 600 pulses). Each patient underwent fMRI pre- and post-iTBS as he or she performed a semantic decision/tone decision (SDTD) task, which has previously been shown to reliably localize residual language areas in patients with aphasia after stroke (Eaton et al., 2008). The authors reported significant improvement on a semantic verbal fluency task, as well as a trend toward improved functional communication, collected by self-report. Recently, Abo et al. (2012) extended this work by defining stimulation sites not only by fMRI activation patterns but also by the type of aphasia. In patients with non-fluent aphasia they applied inhibitory rTMS to either the right or left IFG and in those with fluent aphasia to either the right or left superior temporal gyrus (STG); stimulation application (STG or IFG) was based on fMRI activation patterns during a language task. They observed improvement after 10 daily 1 Hz rTMS sessions (40 min/day) in auditory and reading comprehension and repetition in patients with non-fluent aphasia, and in spontaneous speech in patients with fluent aphasia.

Application of optimal site-finding protocols, either rTMS- or neuroimaging-driven, is likely an improvement over blinded application of therapeutic rTMS as it accounts for individual variability in clinical factors such as lesion size and volume that could differentially influence the mechanisms of neuroplasticity in each patient (Heiss and Thiel, 2006). For example, one site that may be optimal in a patient with a small lesion may not be appropriate for another patient with more extensive damage. The extent of transcallosal disinhibition, or propensity of involvement of the perilesional and potentially beneficial contralateral homologs, differs among patients, and therefore, site-finding protocols help to meet individual treatment needs. One limitation in some of the above studies is a lack of sham or a control group. Without a comparison group in which rTMS was not applied, it is difficult to conclude whether the observed beneficial effects were stimulation-specific or whether they were simply a result of increased general arousal or placebo effect. Additionally, the recently conducted fMRI-driven treatment studies have focused on relatively short outcomes. Thus, the long-term therapeutic benefits of this approach are yet to be explored in large, randomized, and sham-controlled clinical trials (e.g., an ongoing NCT01512264).

Kakuda et al. (2011) recently utilized a novel rTMS approach employing two different frequencies of stimulation. They primed 4 chronic patients with motor-dominant aphasia with 6 Hz rTMS for 10 min before applying the standard 1 Hz rTMS for 20 min for 18 sessions in 11 days. An intensive 60-min speech therapy was also provided to all patients after the TMS protocol. Improvement in several language functions was observed; however, the results differed in each patient with most improvements observed in naming and repetition. Based on prior studies (Iyer et al., 2003; Carey et al., 2010), the authors posited that priming with 6 Hz rTMS would provide “more potent and long-standing suppressive effect” than the more typical 1 Hz rTMS 20 min protocol. However, since the authors did not directly compare effects of a standard approach vs. 6 Hz priming in either this study or a subsequent follow-up study, it is difficult to directly compare their therapeutic effect. Another limitation of this study was lack of long-term follow-up, so persistent effects of this stimulation approach also remain unknown.

While most studies have examined therapeutic effects of rTMS in chronic aphasia, more investigations are beginning to emerge that focus on patients in the sub-acute phase of stroke recovery (Weiduschat et al., 2011; Kindler et al., 2012; Waldowski et al., 2012; Thiel et al., 2013), a period during which spontaneous physiological restoration may still be ongoing. Kindler et al. (2012) assessed the effects of cTBS applied to the right hemisphere Broca's homolog in patients in sub-acute or chronic phases of post-stroke aphasia recovery. They observed that though both patient groups receiving cTBS significantly improved when compared to the sham group, patients in the sub-acute phase were the best responders as tested by timed picture naming accuracy and RT post-cTBS. This finding is crucial as it favorably supports the application of therapeutic rTMS or TBS early on, even sub-acutely. However, yet again, long-term follow-up was not carried out in this study, preventing an assessment of whether this approach had enduring benefits for patients.

Long-lasting effects of inhibitory rTMS have been reported in several studies involving patients with chronic aphasia. For example, Martin et al. (2009) showed that the improvements in picture naming task and phrase length post-rTMS in a chronic patient with non-fluent aphasia lasted for at least 43 months (over 3 and half years). Another study reported the symptomatic benefits post-rTMS lasting up to 12 months (Barwood et al., 2013) as compared to the group that received sham stimulation. Unfortunately, most studies that focused on sub-acute patients have lacked evaluation of long-term benefits. One exception is Waldowski et al. (2012) who reported reduction in aphasia severity 15 weeks post-stimulation in a group of sub-acute patients receiving rTMS as compared to the sham group (Waldowski et al., 2012). However, accuracy in naming improved similarly across both treatment groups, with only a slight benefit in RT in the treatment group. These findings suggest that improvement in some language functions may in fact be non-specific to stimulation. Because there are ongoing physiological neuroplastic changes in the perilesional and homotopic language areas during the acute and subacute phases of post-stroke recovery (Saur et al., 2006), patients are more likely to improve over a course of weeks irrespective of rTMS application. Therefore, more research is necessary to demonstrate long-lasting and stimulation-specific effects of rTMS, especially when applied early after stroke.

Because mechanisms of neuroplasticity may differ as a function of disease duration, it is important that stimulation be delivered in ways that take advantage of and augment the specific neuroplastic changes thought to be at play during the phase of post-stroke recovery in which TMS is being delivered. For example, in some patients who are recruiting the right hemisphere in a compensatory manner in the sub-acute or acute phases of recovery, applying inhibitory rTMS to the right homologs may not be appropriate. In this case, excitatory rTMS of left perilesional language areas (Szaflarski et al., 2011b) may prove more beneficial. Therefore, individual site-finding, driven either by transient rTMS effects or functional neuroimaging, may be the best approach to take while mechanisms of neural recovery remain dynamic. However, this speculation needs to be evaluated in future studies. Future studies of acute and subacute patients should also include multiple long-term follow-ups, in order to better inform the long-term efficacy of these approaches.

### Transcranial direct current stimulation (tDCS)

Recent years have seen a surge of interest in the use of tDCS in order to modulate cognitive function and to improve outcomes in a variety of clinical areas including stroke recovery. Typically tDCS is administered by delivering small electric currents (1–2 mA) to the scalp by a battery-driven device connected to two large (often 5 × 7 cm^2^ or 5 × 5 cm^2^) saline-soaked surface electrodes (Nitsche and Paulus, 2000). Although current flows through both electrodes, by convention the electrode that is being used to target the brain regions to be stimulated is considered the “active” electrode; the other electrode—termed the “reference” or “return” electrode by convention—is typically placed on the supraorbital region (over the forehead) or at a site off the head. Currents delivered during tDCS are not sufficient to generate action potentials, but are sufficient to incrementally alter neuronal resting membrane potentials. Thus, tDCS is often conceptualized as a neuromodulatory rather than a neurostimulatory technique. Like rTMS, tDCS can alter cortical excitability in predictable ways; anodal tDCS (a-tDCS) is believed to increase cortical excitability and cathodal tDCS (c-tDCS) decreases cortical excitability (Nitsche and Paulus, 2000, 2001).

To date, many studies employing tDCS as a therapy for aphasia have adopted approaches that are broadly consistent with an interhemispheric inhibition model of aphasia recovery (Heiss and Thiel, 2006; Hamilton et al., 2011). That is, most investigations have involved either a-tDCS centered on left hemisphere language areas (Baker et al., 2010; Fiori et al., 2011; Fridriksson et al., 2011; Marangolo et al., 2013) in order to either increase the excitability in the perilesional and residual fronto-temporal language areas, or c-tDCS applied to the right hemisphere homotopic areas (Kang et al., 2011) to inhibit over activation (due to transcollasal disinhibition) in the contralesional right homologs. Most of these studies applied 1–2 mA of current for 10–30 min over 5–10 days in patients with chronic aphasia. Several studies compared the therapeutic effects of a- or c-tDCS to sham treatment in a within-subject and cross-over design and provided concurrent speech and language training.

Outcome measures consisted of aphasia severity scales [WAB, Battery for the Analysis of Aphasic Disorders (BADA)] and language batteries including picture naming tasks such as the BNT. Monti et al. (2008) were first to report transient effects of tDCS in patients with aphasia. In their study 8 patients with chronic aphasia received sham or active tDCS at 2 mA for 10 min over the left frontotemporal area; 4 received a-tDCS and sham, while the other 4 received c-tDCS and sham (Monti et al., 2008). Interestingly, picture naming accuracy improved after 10 min of c-tDCS, while a-tDCS and sham induced no changes in naming performance. This finding is counterintuitive as it suggests that inhibiting the damaged left hemispheric language network may improve language functions. The authors argued that the mechanism of improvement may be the stimulation-specific inhibition of overactive interneurons in the damaged left hemisphere. However, these findings have not yet been replicated in an intervention study with multiple tDCS sessions and in blinded patients/evaluators.

Naming accuracy also improved in 10 chronic aphasic patients, who received 1 mA a-tDCS on intact left frontal areas for 20 min per day for 5 days (Baker et al., 2010); they also received sham in the same manner, where application of active and sham tDCS was randomized. The active site of stimulation was determined individually by examining fMRI activation patterns during a naming task. In a follow-up study, Fridriksson et al. (2011) applied a similar stimulation and site-finding protocol in 8 chronic patients with fluent aphasia and reported improvement in naming reaction times after a-tDCS as compared to sham. In addition, they reported that these RT benefits persisted at least 3 weeks after stimulation. Both these findings are in line with the notion that perilesional recruitment is necessary for post-stroke aphasia recovery; the beneficial effects of tDCS are presumed to be mediated by enhanced activity of residual left hemisphere language areas as well as compensatory functional changes in left hemisphere perilesional areas.

As noted above, the interhemispheric inhibition model of post-stroke language plasticity posits that activity of right hemisphere structures may interfere with the compensatory recovery of left hemisphere perilesional area. Although this model has underpinned the approach of numerous investigators, Kang et al. (2011) is the only group to date to apply inhibitory right c-tDCS to patients with chronic aphasia with the aim of inhibiting the potentially deleterious right hemisphere activity (Kang et al., 2011). In a cross-over design, Kang et al. demonstrated that picture naming accuracy improved after c-tDCS on right frontal areas compared to sham in 10 chronic patients. In another study, Floel et al. (2011) applied excitatory a-tDCS as well as inhibitory c-tDCS and sham over the right temporo-parietal cortex during anomia training (Floel et al., 2011). The aim was to demonstrate enhancing therapeutic effects of anomia training in context of inhibitory vs. excitatory tDCS of the areas in the right hemisphere. Naming ability improved across all three groups with anomia training, and the effects lasted for at least 2 weeks. Interestingly, a-tDCS, and not c-tDCS, of the right hemispheric exhibited greater and longer-lasting improvement in the naming ability, as compared to sham. This finding suggests that stimulating right homotopic areas may be more reliable in enhancing effects of anomia training than inhibiting them. Beneficial role of right hemispheric areas in language improvement with tDCS is highlighted in this study.

Recently, Marangolo et al. (2013) took a different approach to language training and outcome measures where they assessed whether enhancing activity in the left language areas by a-tDCS can improve informative or pragmatic speech, rather than focusing on improvement on neuropsychological assessments alone (Marangolo et al., 2013). They used different video-clips describing “everyday life situations” for training and testing 12 chronic patients with aphasia who underwent a-tDCS and sham centered on left Broca's and left Wernicke's areas in a cross-over design. The authors reported improved language performance in terms of increased use of content-units and increased verb and sentence production after a-tDCS on Broca's area as compared to Wernicke's and sham tDCS. Additionally, the effects sustained for at least a month after the treatment ended. Sustained improvement in an ecologically valid measure in this study is promising, in that tDCS paired with language training may be able to improve the overall ability of patients with aphasia to communicate in everyday life.

While almost all tDCS intervention studies focused on chronic patients, You et al. (2011) studied effects of tDCS on sub-acute patients with global aphasia (You et al., 2011). Rather than a cross-over design commonly adopted by other investigators, You et al. included a separate sham control group. Of 21 patients, 7 patients received a-tDCS centered on left STG (Wernicke's), 7 received c-tDCS on right STG and 7 received sham stimulation, for 30 min a day for 10 days. During stimulation (sham and active), patients underwent speech and language therapy. As predicted with stimulation of temporal language areas (regions broadly involved in language comprehension), the authors reported that auditory verbal comprehension improved significantly more in patients receiving right c-tDCS (inhibitory) compared to those receiving left a-tDCS (excitatory) or sham stimulation. In addition, across active and sham groups, improvement was observed in the aphasia severity scale and in spontaneous speech. This finding is similar to (Waldowski et al., 2012) who applied rTMS in subacute patients and observed across group (sham and active) improvements. It appears that these non-stimulation specific improvements may be typical in sub-acute patients undergoing spontaneous restitution of language functions. Therefore, carefully chosen neuropsychological assessments and inclusion of a sham group are required to establish therapeutic benefits of tDCS in patients who have dynamic rather than static aphasia. Of importance is the finding that inhibition of right temporal language areas, rather than excitatory left stimulation common among other studies involving chronic patients, resulted in improved language functions. These findings suggest that during sub-acute recovery stages, inhibition of right hemisphere activity may be an effective therapeutic approach which is consistent with the trajectory of neuroimaging changes associated with acute, sub-acute, and chronic post-stroke recovery as demonstrated by Saur et al. (2006).

Overall, a review of tDCS intervention studies in post-stroke aphasia reveals that while some studies have made efforts to increase their sample sizes (up to 14 patients) and use ecologically valid outcome measure, several parameters in study design and stimulation methodology could be improved. First, except one study (Marangolo et al., 2013), most current intervention studies did not include a long-term follow-up to address the long-lasting benefits of tDCS in improving language functions in post-stroke aphasia. Secondly, the effects of tDCS in the sub-acute phase of recovery need more consideration. For example, studies comparing the therapeutic effects of a particular electrode montage (c-tDCS vs. a-tDCS on the right or left language areas) in sub-acute vs. chronic patients are desirable. Based on the effects of disease duration and lesion size and location on the models of neuroplasticity, a mechanistic approach to tDCS electrode montage may be more appropriate. This can be achieved by a multimodal neuroimaging-driven tDCS intervention (Hunter et al., 2013), where the placement of active electrodes in the left or right hemispheres can be based on the fMRI activation patterns. Additionally, a dual-hemispheric tDCS approach i.e., simultaneous right inhibitory and left excitatory tDCS, may also prove beneficial. Evidence of changes in the underlying brain activity as a function of therapeutic tDCS or a particular montage of tDCS has not yet been carried out in patients with aphasia. Improvement in language functions may be directly related to induced neuroplasticity; however, as the current literature stands, there is no evidence of this association in post-stroke aphasia.

As far as tDCS methodologies are concerned, new evidence suggests that c-tDCS does not reliably decrease the underlying cortical excitability. Depending on the duration (>15 min) and intensity of stimulation (>1 mA), c-tDCS may behave more like a-tDCS, in that it increases, rather than decreasing, the cortical excitability (Batsikadze et al., 2013). Specifically, Batsikadze et al. showed that 2 mA c-tDCS, when applied for 20 min on the motor cortex, induced cortical excitation, rather than inhibition, in healthy individuals. While 1 mA c-tDCS for the same duration and site induced cortical inhibition. Interestingly, at least one study (Monti et al., 2008) reported transient beneficial effects of 2 mA 10 min c-tDCS on the lesioned left hemisphere. Speculatively, these effects may have resulted from cortical excitation by c-tDCS of the perilesional or residual language areas, and not cortical inhibition as previously believed. However, effects of c-tDCS in stroke patients, particularly when applied to the lesioned hemisphere, may be different than in healthy individuals. Because of the loss of typical cortical structure, the flow of current in the lesioned hemisphere may be quite diverse. In contrast to Monti et al. (2008), Kang et al. (2011), and You et al. (2011) applied c-tDCS to the unaffected right homologs of the STG and the Broca's area, respectively. Interestingly, this approach also led to improved language functions; c-tDCS applied to the right STG was more beneficial than a-tDCS applied to the left STG and more than sham (You et al., 2011). However, since these studies did not provide a model of current flow (Datta et al., 2009, 2010), nor followed-up with a measure of changes in cortical excitability, it is difficult to infer the mechanism that underlies the observed improvement. It is clear, however, that a multimodal approach to tDCS is required for understanding how different tDCS parameters, especially c-tDCS, remodel the bilateral language networks to enable recovery. Future studies should describe a relationship between tDCS “dose and response,” to translate its application as an effective treatment for post-stroke aphasia.

## Neuroplasticity induced by non-invasive brain stimulation

In this section, we will discuss the observed changes in the brain activity induced by NBS. We reviewed only those studies that paired direct measures of neuroplasticity (PET, fMRI, EEG-ERP, etc.) after therapeutic rTMS as no such studies have yet been carried out with tDCS (summary in Table 1). Based on neuroimaging accounts of neuroplasticity in spontaneous recovery described earlier, we expect that sparing of key language areas in the left hemisphere and an increased contribution of the left hemispheric residual and perilesional areas after therapeutic rTMS would be associated with improved language functions. However, just as we discussed in the case of spontaneous neuroplasticity, the contributions of right hemisphere homologs to stimulation-driven neuroplasticity is not clear.

In one study, Martin et al. (2009) suppressed right PTr in a chronic non-fluent aphasic patient over 10 daily 1 Hz rTMS sessions to induce long-lasting improvement in naming and propositional speech (Martin et al., 2009). Importantly, at 16 and 43 months after rTMS, fMRI activation patterns revealed a greater recruitment of perilesional (left)/perisylvian areas, specifically the left supplementary motor area and the left and right sensorimotor mouth areas, and areas along the fronto-temporal language network. Activation of the right IFG, observed pre-TMS persisted at 16 months after stimulation. Overall, the authors argued that the increased activation in the bilateral motor and perilesional language areas after repetitive suppression of right PTr revealed a leftward activation shift supported by improvement in language functions. However, the contribution from changes in the right IFG to improved performance after rTMS is unclear. Perhaps, suppressing an inefficient node i.e., the right PTr (Turkeltaub et al., 2011) enabled reorganization of the bilateral functional networks by increasing left hemispheric recruitment as well as refining the beneficial role of right homologs. In contrast, fMRI activation patterns in a second patient, also suffering from chronic non-fluent aphasia, did not show an increased left hemispheric recruitment post-rTMS at 3 or 6 months, and language performance on naming and propositional speech did not improve in this patient (right IFG activation was consistently observed in this patient). In this patient, a larger frontal and temporal lesion and subcortical white matter damage may have been associated with more severe non-fluent aphasia and lack of improvement post-rTMS. The authors argued that since key language regions in the left hemisphere were not spared in this patient, suppression of right PTr by rTMS could not promote recovery. We posit that lesion extent and location may have impeded induced reorganization in the bilateral functional network with rTMS (Heiss and Thiel, 2006).

A different case study by Turkeltaub et al. (2012) shed some light on the role of right hemispheric homotopic areas (Turkeltaub et al., 2012). A right hemispheric stroke in a patient with chronic non-fluent aphasia, who underwent therapeutic rTMS (10 daily 1 Hz rTMS sessions) after an initial left hemispheric stroke, worsened her aphasia symptoms. After her left stroke, repetitive inhibition of right PTr induced improvement in her language functions for up to 2 months. FMRI activation patterns obtained on the first day of rTMS, before and after treatment, indicated that right PTr activity was in fact suppressed but the expected increase in left hemispheric activity was not yet present. However, within 3 months of therapeutic rTMS, she suffered from a second stroke, this time on the right side of the brain, which worsened her language functions. At 3 months after her second stroke, language functions were decreased more than other cognitive functions. This case provided authors with a unique opportunity to examine the contribution of right homolog damage to the overall language functions after initial dominant hemispheric stroke. If one assumes that recruitment of all homotopic right areas is deleterious to recovery, then in this case the language functions should not have been affected or could have improved. Therefore, consistent with the notion that the “right hemisphere can speak” (Code, 1997) the authors suggest that right hemisphere homotopic areas may support functional recovery.

In contrast to the above studies, Weiduschat et al. (2011) demonstrated that PET activation patterns during a verb-generation task were suppressed in the right hemisphere post-rTMS (compared to pre-TMS), while they were significantly more prominent in the right hemisphere in the sham treatment group (Weiduschat et al., 2011). In this study, 1 Hz rTMS was applied either to Broca's homolog in the right hemisphere (right PTr) followed by speech and language therapy or to control area (vertex); significant improvements in language functions were observed in 6 patients receiving rTMS compared to 4 control patients. Since enrolled patients were in the sub-acute phase of recovery there was a tendency in the control patients to recruit right homologs consistent with the result of the study by Saur et al. (2006). In patients receiving rTMS, right hemispheric involvement was suppressed, presumably by functional inhibition of right PTr, which may have contributed to the observed improvements in this group. Thus, the increased right hemispheric involvement in the absence of right PTr suppression in the control group may represent an inefficient mode of recovery. We posit that inefficient activation of specific sites in the right hemisphere (right PTr) during spontaneous course of recovery may be detrimental to the recruitment of perilesional and residual language areas in favor of recovery. Therefore, suppressing activity in the right PTr may induce neuroplastic changes characterized by release in activation of an inefficient node and thus, promoting recruitment of left hemispheric areas. However, an important caveat in this study is a lack of association between changes in PET activation patterns and language improvements; this may be, in part, related to a small number of subjects enrolled in this study and/or high drop-out rate (4/14). This last shortcoming was recently addressed by Thiel et al. (2013) in a larger group of 24 sub-acute stroke patients with different aphasia types (non-fluent, fluent, global, and amnestic). After 10 sessions of 1 Hz rTMS combined with 45 min of speech and language therapy, 13 patients in the rTMS group showed improvement on the AAT, a comprehensive aphasia severity scale, with largest improvement in naming subtest as compared to the 11 patients in the sham group (Thiel et al., 2013). Relevant to prior findings, a change in bilateral functional activity was observed in this study. Activation volume index, a measure of change in the volume of activation (significant voxels) between left and right hemispheres, revealed increased PET activation in the left hemisphere in the rTMS compared to the sham group; similar right hemispheric activation was observed pre-rTMS between sham and rTMS groups. Importantly, the level of change in activation followed a linear relationship with the change in AAT scores i.e., greater activation shift toward left hemisphere was associated with greater improvement on AAT scores after rTMS. The findings of this study suggest that induced cortical reorganization of language functions to the left hemisphere relate to the improvements after therapeutic rTMS.

Direct measurement of activation changes in the language areas before and after rTMS was also carried out by Szaflarski et al. (2011b) in chronic patients (Szaflarski et al., 2011b). This study differed from other rTMS aphasia intervention studies in 2 important ways: (1) rather than suppressing areas in the right hemisphere, residual left perisylvian areas were stimulated in an excitatory stimulation protocol, and (2) instead of standard continuous delivery of rTMS, iTBS was applied (for more information refer to Table 1). Repeated iTBS showed significant improvement in 6 out of 8 patients in a semantic fluency task. Importantly, fMRI activation patterns post-iTBS revealed increased recruitment of perilesional fronto-temporo-parietal areas, as well as a shift in activation toward left frontal and temporal language areas. Recruitment of some right hemispheric subcortical and motor areas was also observed. The findings in this study corroborate with those in other studies using inhibitory rTMS, and suggest that recruitment of perilesional areas as well as reduction in inefficient activation of right homotopic sites may subserve improved language functions.

In addition to functional changes in activation patterns after rTMS, white matter structural integrity may also improve. In a follow-up study, (Allendorfer et al., 2012b) examined whether iTBS administered in the earlier study could also potentially improve structural white matter integrity, specifically in the areas that showed greater fMRI activation with iTBS (Szaflarski et al., 2011b; Allendorfer et al., 2012b). They used DTI, and compared fractional anisotropy (FA) changes pre- vs. post-iTBS in the same group of chronic patients as in (Szaflarski et al., 2011b) DTI-FA provides a measure of white matter integrity and directionality by indexing restricted diffusion of water molecules in different tissue types (Basser and Pierpaoli, 1996; Pierpaoli et al., 1996; Bennett et al., 2010; Allendorfer et al., 2012b); higher FA values correspond to greater white matter integrity. Increases in FA values were observed post-iTBS compared to pre-iTBS in left hemispheric areas close to the stimulation site and also near the regions that showed greater fMRI activation in the earlier study. Specifically, measurable increases in FA were observed in the left IFG, anterior cingulate, insula and right temporal and parietal areas, along with bilateral increases in the posterior cingulate. However, improvement on language performance (semantic fluency task) did not correlate with changes in FA probably because of the relatively low sample size (*N* = 8). Nonetheless, the observed changes in the white matter integrity in the left perilesional areas as well as in some right hemisphere areas present a similar pattern to changes in functional activation. Future studies with a sham-controlled arm in a larger group of patients will validate or disprove these findings (NCT01512264).

Additionally, electrophysiologic changes after therapeutic rTMS have also been reported. Event-related potentials (ERP) derived from EEG can also characterize induced neuroplasticity in patients with aphasia as demonstrated by changes related to speech-language therapies (Pulvermuller et al., 2005; Laganaro et al., 2008; Barwood et al., 2011b) and pharmacological treatments (Szelies et al., 2001). Recently, Barwood et al. (2011a,b) examined the effects of 1 Hz rTMS on right PTr in 12 patients with chronic non-fluent or global aphasia (Barwood et al., 2011a,b,c). At 2 months after stimulation, 6 patients who received rTMS for 20 min/day for 10 days improved significantly more than 6 patients who received sham treatment of same duration; specifically naming, expressive language, and auditory comprehension improved. Further, in a follow-up study, Barwood et al. (2011b) reported stimulation-specific effects on N400, an ERP component time-locked to semantic language processing. Specifically, at 2 months the overall mean amplitude, peak amplitude, and areas under the curve of the N400 component were significantly higher in the treated group than in the sham group; higher amplitudes reflect improvement in the language function, in this case on a semantic-lexical task. Interestingly, in this group of patients, transient changes (increases) on N400 parameters were not found, meaning ERP amplitudes at baseline vs. 1 week after rTMS were not different. The authors speculated that rTMS-specific modulation in the bilateral language network supported by increase in N400 component post-rTMS may be crucial for the improvements observed over time. Additionally, the findings suggest that rTMS-induced neuroplasticity may be time-dependent such that reorganization in the bilateral language network may require protracted time to materialize.

This observation of time-dependency of induced neuroplasticity after therapeutic rTMS reveals one recurring limitation of studies reviewed in this section. Most of these studies lacked a long-term follow-up i.e., these studies reported changes in activation patterns acutely after rTMS, but unlike Martin et al. (2009) and Barwood et al. (2011b), failed to report sustained changes in activation patterns a few months or years after treatment. This information is critical as it will inform us about the direct effects of rTMS on long-term neuroplasticity.

## Safety of non-invasive brain stimulation

The most serious, albeit unlikely, health risk associated with TMS is induction of a seizure (Homberg and Netz, 1989; Kandler, 1990). In the years since induced seizures were first observed in association with TMS, rigorous safety guidelines have been developed which specify the number of pulses that may safely be given as a function of stimulus intensity (% of Motor Evoked Potential), frequency, and inter-train interval (Wasserman, 1998; Bolognini et al., 2009; Rossi et al., 2009). Numerous subsequent studies of rTMS have demonstrated that stimulation within these parameters is safe in normal persons, patients with stroke (Hao et al., 2013), and even epilepsy patients (Bae et al., 2007). Administration of TBS within published parameters has been well tolerated in healthy adult studies; only one study reported a seizure in a healthy subject caused by TBS used with intensity set at 100% of the RMT (Oberman and Pascual-Leone, 2009), greater than the 80% of active threshold typically used in recent studies (Huang et al., 2005; Szaflarski et al., 2011b). Thus, there has been no convincing evidence that rTMS performed within established guidelines or TBS performed using published parameters can cause short- or long-term seizures/epilepsy or other ill effects.

To date, there have been no reports of seizures or other short- or long-term severe adverse events related to the use of tDCS. Several recent studies have reported mild side effects of tDCS in both healthy individuals (Brunoni et al., 2011; Kessler et al., 2012) and patient populations (Poreisz et al., 2007) including itching, tingling, burning, pain, and headaches, which were not long-lasting.

## Future directions

Neurorehabilitation of post-stroke aphasia with the use NBS shows a lot of promise. Throughout this review, we have highlighted several advantages as well as limitations of current NBS methodologies and study design in an attempt to advance its use as an effective tool for the treatment of post-stroke aphasia. One overarching goal of future studies should be to capture therapeutic benefits of NBS not only on neuropsychological language batteries but also on everyday communication abilities.

Underlying recovery mechanisms and neuroplasticity with NBS in post-stroke aphasia still remain an open question. Recovering language networks are dynamic depending on multiple factors including the location of the lesion and its size, time since injury, intensity and type of provided intervention, age at the time of injury and handedness. There is an agreement in the field about the beneficial role of left hemispheric perilesional and residual language areas in both spontaneous as well induced with NBS recovery. However, the debate on the role of right hemispheric homotopic areas continues. Several investigators concur that recruitment of areas in the right hemisphere is an inefficient mode of recovery in patients with aphasia while some argue that rather than all areas, recruitment of some specific site(s) in the right hemisphere may be inefficient or deleterious to recovery. In future studies, we recommend parsing out specific functions of right hemispheric homotopic areas during spontaneous recovery, and persisting activity in some right hemispheric areas in recovery induced with NBS. Future studies should closely address the individual determinants of patterns of neuroplastic changes both to guide NBS treatment and to assess functional recovery as well as the role of neuronavigation with TMS, fMRI, PET or other techniques. In particular, future multimodal approaches pairing neuroimaging and electrophysiological measures with therapeutic NBS will more clearly define its potential in aiding rehabilitation after an aphasia-producing stroke.

### Conflict of interest statement

The authors declare that the research was conducted in the absence of any commercial or financial relationships that could be construed as a potential conflict of interest.

## References

[B1] Abo M. Kakuda W. Watanabe M. Morooka A. Kawakami K. Senoo A. (2012). Effectiveness of low-frequency rTMS and intensive speech therapy in poststroke patients with aphasia: a pilot study based on evaluation by fMRI in relation to type of aphasia. Eur. Neurol. 68, 199–208 10.1159/000338773 22948550

[B2] Allendorfer J. B. Kissela B. M. Holland S. K. Szaflarski J. P. (2012a). Different patterns of language activation in post-stroke aphasia are detected by overt and covert versions of the verb generation fMRI task. Med. Sci. Monit. 18, CR135–CR147 10.12659/MSM.882518 22367124 PMC3319663

[B3] Allendorfer J. B. Storrs J. M. Szaflarski J. P. (2012b). Changes in white matter integrity follow excitatory rTMS treatment of post-stroke aphasia. Restor. Neurol. Neurosci. 30, 103–113 10.3233/RNN-2011-0627 22233802 PMC3316910

[B4] Bae E. H. Schrader L. M. Machii K. Alonso-Alonso M. Riviello J. J. Pascual-Leone A. (2007). Safety and tolerability of repetitive transcranial magnetic stimulation in patients with epilepsy: a review of the literature. Epilepsy Behav. 10, 521–528 10.1016/j.yebeh.2007.03.004 17493877

[B5] Baker J. M. Rorden C. Fridriksson J. (2010). Using transcranial direct-current stimulation to treat stroke patients with aphasia. [Clinical Trial Research Support, N.I.H., Extramural]. Stroke 41, 1229–1236 10.1161/STROKEAHA.109.576785 20395612 PMC2876210

[B6] Barwood C. H. S. Murdoch B. E. Riek S. O'Sullivan J. D. Wong A. Lloyd D. (2013). Long term language recovery subsequent to low frequency rTMS in chronic non-fluent aphasia. NeuroRehabilitation 32, 915–928 10.3233/NRE-130915 23867417

[B7] Barwood C. H. S. Murdoch B. E. Whelan B. M. Lloyd D. Riek S. O'Sullivan J. (2011a). The effects of low frequency Repetitive Transcranial Magnetic Stimulation (rTMS) and sham condition rTMS on behavioural language in chronic non-fluent aphasia: short term outcomes. NeuroRehabilitation 28, 113–128 10.3233/NRE-2011-0640 21447912

[B8] Barwood C. H. S. Murdoch B. E. Whelan B. M. Lloyd D. Riek S. O'Sullivan J. D. (2011b). Modulation of N400 in chronic non-fluent aphasia using low frequency Repetitive Transcranial Magnetic Stimulation (rTMS). Brain Lang. 116, 125–135 10.1016/j.bandl.2010.07.004 20678791

[B9] Barwood C. H. S. Murdoch B. E. Whelan B. M. Lloyd D. Riek S. Sullivan J. D. O. (2011c). Improved language performance subsequent to low-frequency rTMS in patients with chronic non-fluent aphasia post-stroke. Eur. J. Neurol. 18, 935–943 10.1111/j.1468-1331.2010.03284.x 21138505

[B10a] Barwood C. H. S. Murdoch B. E. Whelan B. M. Lloyd D. Riek S. O'Sullivan J. D. (2012). Improved receptive and expressive language abilities in nonfluent aphasic stroke patients after application of rTMS: An open protocol case series. Brain Stimulation 5, 274–286 10.1016/j.brs.2011.03.005 22037124

[B10] Basser P. J. Pierpaoli C. (1996). Microstructural and physiological features of tissues elucidated by quantitative-diffusion-tensor MRI. J. Magn. Reson. B 111, 209–219 10.1006/jmrb.1996.0086 8661285

[B11] Bates E. D'Amico S. Jacobsen T. Szekely A. Andonova E. Devescovi A. (2003). Timed picture naming in seven languages. Psychon. Bull. Rev. 10, 344–380 10.3758/BF03196494 12921412 PMC3392189

[B12] Batsikadze G. Moliadze V. Paulus W. Kuo M. F. Nitsche M. A. (2013). Partially non-linear stimulation intensity-dependent effects of direct current stimulation on motor cortex excitability in humans. J. Physiol. 591, 1987–2000 10.1113/jphysiol.2012.249730 23339180 PMC3624864

[B13] Belin P. VanEeckhout P. Zilbovicius M. Remy P. Francois C. Guillaume S. (1996). Recovery from nonfluent aphasia after melodic intonation therapy: a PET study. Neurology 47, 1504–1511 10.1212/WNL.47.6.1504 8960735

[B14] Bennett I. J. Madden D. J. Vaidya C. J. Howard D. V. Howard J. H. (2010). Age-related differences in multiple measures of white matter integrity: a diffusion tensor imaging study of healthy aging. Hum. Brain Mapp. 31, 378–390 10.1002/hbm.20872 19662658 PMC2826569

[B15] Berthier M. L. (2005). Poststroke aphasia - epidemiology, pathophysiology and treatment. Drugs Aging 22, 163–182 10.2165/00002512-200522020-00006 15733022

[B16] Bolognini N. Pascual-Leone A. Fregni F. (2009). Using non-invasive brain stimulation to augment motor training-induced plasticity. J. Neuroeng. Rehabil. 6, 8–20 10.1186/1743-0003-6-8 19292910 PMC2667408

[B17] Brady K. H. Enderby P. (2010). Speech and language therapy for aphasia following stroke. Cochrane Database Syst. Rev. 1–166 10.1002/14651858.CD000425.pub2 20464716

[B18] Brunoni A. R. Amadera J. Berbel B. Volz M. S. Rizzerio B. G. Fregni F. (2011). A systematic review on reporting and assessment of adverse effects associated with transcranial direct current stimulation. Int. J. Neuropsychopharmacol. 14, 1133–1145 10.1017/S1461145710001690 21320389

[B19] Carey J. R. Anderson D. C. Gillick B. T. Whitford M. Pascual-Leone A. (2010). 6-Hz primed low-frequency rTMS to contralesional M1 in two cases with middle cerebral artery stroke. Neurosci. Lett. 469, 338–342 10.1016/j.neulet.2009.12.023 20026185 PMC2815205

[B20] Code C. (1997). Can the right hemisphere speak? Brain Lang. 57, 38–59 10.1006/brln.1997.1833 9126406

[B21] Cornelissen K. Laine M. Tarkiainen A. Jarvensivu T. Martin N. Salmelin R. (2003). Adult brain plasticity elicited by anomia treatment. J. Cogn. Neurosci. 15, 444–461 10.1162/089892903321593153 12729495

[B22a] Cotelli M. Fertonani A. Miozzo A. Rosini S. Manenti R. Padovani A. (2011). Anomia training and brain stimulation in chronic aphasia. Neuropsychol. Rehabil. 21, 717–741 10.1080/09602011.2011.621275 22011016

[B22] Datta A. Bansal V. Diaz J. Patel J. Reato D. Bikson M. (2009). Gyri-precise head model of transcranial direct current stimulation: improved spatial focality using a ring electrode versus conventional rectangular pad. Brain Stimul. 2, 201–207 10.1016/j.brs.2009.03.005 20648973 PMC2790295

[B23] Datta A. Bikson M. Fregni F. (2010). Transcranial direct current stimulation in patients with skull defects and skull plates: high-resolution computational FEM study of factors altering cortical current flow. Neuroimage 52, 1268–1278 10.1016/j.neuroimage.2010.04.252 20435146 PMC2910315

[B24] Eaton K. P. Szaflarski J. P. Altaye M. Ball A. L. Kissela B. M. Banks C. (2008). Reliability of fMRI for studies of language in post-stroke aphasia subjects. Neuroimage 41, 311–322 10.1016/j.neuroimage.2008.02.033 18411061 PMC2474692

[B25] Eliassen J. C. Boespflug E. L. Lamy M. Allendorfer J. Chu W. J. Szaflarski J. P. (2008). Brain-mapping techniques for evaluating poststroke recovery and rehabilitation: a review. Top. Stroke Rehabil. 15, 427–450 10.1310/tsr1505-427 19008203 PMC2663338

[B26] Fiori V. Coccia M. Marinelli C. V. Vecchi V. Bonifazi S. Ceravolo M. G. (2011). Transcranial direct current stimulation improves word retrieval in healthy and nonfluent aphasic subjects. J. Cogn. Neurosci. 23, 2309–2323 10.1162/jocn.2010.21579 20946060

[B27] Fitzgerald P. B. Fountain S. Daskalakis Z. J. (2006). A comprehensive review of the effects of rTMS on motor cortical excitability and inhibition. Clin. Neurophysiol. 117, 2584–2596 10.1016/j.clinph.2006.06.712 16890483

[B28] Floel A. Meinzer M. Kirstein R. Nijhof S. Deppe M. Knecht S. (2011). Short-term anomia training and electrical brain stimulation. Stroke 42, 2065–2067 10.1161/STROKEAHA.110.609032 21636820

[B29] Fridriksson J. Richardson J. D. Baker J. M. Rorden C. (2011). Transcranial direct current stimulation improves naming reaction time in fluent aphasia a double-blind, sham-controlled study. Stroke 42, 819–821 10.1161/STROKEAHA.110.600288 21233468 PMC8210639

[B30] Hamilton R. H. Chrysikou E. G. Coslett B. (2011). Mechanisms of aphasia recovery after stroke and the role of noninvasive brain stimulation. Brain Lang. 118, 40–50 10.1016/j.bandl.2011.02.005 21459427 PMC3109088

[B31] Hamilton R. H. Sanders L. Benson J. Faseyitan O. Orise C. Naeser M. (2010). Stimulating conversation: enhancement of elicited propositional speech in a patient with chronic non-fluent aphasia following transcranial magnetic stimulation (vol 113, pg 45, 2010). Brain Lang. 113, 101 10.1016/j.bandl.2010.03.004 20159655 PMC2909623

[B32] Hao Z. Wang D. Zeng Y. Liu M. (2013). Repetitive transcranial magnetic stimulation for improving function after stroke. Cochrane Database Syst. Rev. 5, CD008862 10.1002/14651858.CD008862.pub2 23728683 PMC6464739

[B33] Heiss W. D. Kessler J. Thiel A. Ghaemi M. Karbe H. (1999). Differential capacity of left and right hemispheric areas for compensation of poststroke aphasia. Ann. Neurol. 45, 430–438 10.1002/1531-8249(199904)45:4<430::AID-ANA3>3.0.CO;2-P 10211466

[B34] Heiss W. D. Thiel A. (2006). A proposed regional hierarchy in recovery of post-stroke aphasia. Brain Lang. 98, 118–123 10.1016/j.bandl.2006.02.002 16564566

[B35] Homberg V. Netz J. (1989). Generalized seizures induced by transcranial magnetic stimulation of motor cortex. Lancet 2, 1223–1223 10.1016/S0140-6736(89)91835-7 2572937

[B36] Huang Y. Z. Edwards M. J. Rounis E. Bhatia K. P. Rothwell J. C. (2005). Theta burst stimulation of the human motor cortex. Neuron 45, 201–206 10.1016/j.neuron.2004.12.033 15664172

[B37] Hunter M. A. Coffman B. A. Trumbo M. C. Clark V. P. (2013). Tracking the neuroplastic changes associated with transcranial direct current stimulation: a push for multimodal imaging. Front. Hum. Neurosci. 7:495 10.3389/fnhum.2013.00495 23986681 PMC3753560

[B38] Iyer M. B. Schleper N. Wassermann E. M. (2003). Priming stimulation enhances the depressant effect of low-frequency repetitive transcranial magnetic stimulation. J. Neurosci. 23, 10867–10872 14645480 10.1523/JNEUROSCI.23-34-10867.2003 PMC6740990

[B39] Kakuda W. Abo M. Momosaki R. Morooka A. (2011). Therapeutic application of 6-Hz-primed low-frequency rTMS combined with intensive speech therapy for post-stroke aphasia. Brain Inj. 25, 1242–1248 10.3109/02699052.2011.608212 21902549

[B40] Kandler R. (1990). Safety of transcranial magnetic stimulation. Lancet 335, 469–470 10.1016/0140-6736(90)90696-3 1968184

[B41] Kang E. K. Kim Y. K. Sohn H. M. Cohen L. G. Paik N. J. (2011). Improved picture naming in aphasia patients treated with cathodal tDCS to inhibit the right Broca's homologue area. Restor. Neurol. Neurosci. 29, 141–152 10.3233/RNN-2011-0587 21586821 PMC4886370

[B42] Karbe H. Thiel A. Weber-Luxenburger G. Herholz K. Kessler J. Heiss W. D. (1998a). Brain plasticity in poststroke aphasia: what is the contribution of the right hemisphere? Brain Lang. 64, 215–230 10.1006/brln.1998.1961 9710490

[B43] Karbe H. Thiel A. Weber-Luxenburger G. Kessler J. Herholz K. Heiss W. D. (1998b). Reorganization of the cerebral cortex in post stroke aphasia studied with positron emission tomography. Neurology 50, A321–A321

[B44] Kessler S. K. Turkeltaub P. E. Benson J. G. Hamilton R. H. (2012). Differences in the experience of active and sham transcranial direct current stimulation. Brain Stimul. 5, 155–162 10.1016/j.brs.2011.02.007 22037128 PMC3270148

[B45] Kindler J. Schumacher R. Cazzoli D. Gutbrod K. Koenig M. Nyffeler T. (2012). Theta burst stimulation over the right Broca's homologue induces improvement of naming in aphasic patients. Stroke 43, U2175–U2270 10.1161/STROKEAHA.111.647503 22581821

[B46] Laganaro M. Morand S. Schwitter V. Zimmermann C. Schnider A. (2008). Normalisation and increase of abnormal ERP patterns accompany recovery from aphasia in the post-acute stage. Neuropsychologia 46, 2265–2273 10.1016/j.neuropsychologia.2008.02.013 18406433

[B47] Lazar R. M. Speizer A. E. Festa J. R. Krakauer J. W. Marshall R. S. (2008). Variability in language recovery after first-time stroke. J. Neurol. Neurosurg. Psychiatry 79, 530–534 10.1136/jnnp.2007.122457 17846113

[B48] Lee R. G. Vandonkelaar P. (1995). Mechanisms underlying functional recovery following stroke. Can. J. Neurol. Sci. 22, 257–263 8599767 10.1017/s0317167100039445

[B49] Marangolo P. Fiori V. Calpagnano M. Campana S. Razzano C. Caltagirone C. (2013). tDCS over the left inferior frontal cortex improves speech production in aphasia. Front. Hum. Neurosci. 7:539 10.3389/fnhum.2013.00539 24046740 PMC3764371

[B50] Martin P. I. Naeser M. A. Ho M. Doron K. W. Kurland J. Kaplan J. (2009). Overt naming fMRI pre- and post-TMS: two nonfluent aphasia patients, with and without improved naming post-TMS. Brain Lang. 111, 20–35 10.1016/j.bandl.2009.07.007 19695692 PMC2803355

[B51] Medina J. Norise C. Faseyitan O. Coslett H. B. Turkeltaub P. E. Hamilton R. H. (2012). Finding the right words: transcranial magnetic stimulation improves discourse productivity in non-fluent aphasia after stroke. Aphasiology 26, 1153–1168 10.1080/02687038.2012.710316 23280015 PMC3532848

[B52] Monti A. Cogiamanian F. Marceglia S. Ferrucci R. Mameli F. Mrakic-Sposta S. (2008). Improved naming after transcranial direct current stimulation in aphasia. J. Neurol. Neurosurg. Psychiatry 79, 451–453 10.1136/jnnp.2007.135277 18096677

[B53] Musso M. Weiller C. Kiebel S. Muller S. P. Bulau P. Rijntjes M. (1999). Training-induced brain plasticity in aphasia. Brain 122, 1781–1790 10.1093/brain/122.9.1781 10468516

[B54] Naeser M. A. Martin P. I. Baker E. H. Hodge S. M. Czerzenie S. E. Nicholas M. (2004). Overt propositional speech in chronic nonfluent aphasia studied with the dynamic susceptibility contrast fMRI method. Neuroimage 22, 29–41 10.1016/j.neuroimage.2003.11.016 15109995

[B55] Naeser M. A. Martin P. I. Nicholas M. Baker E. H. Seekins H. Kobayashi M. (2005). Improved picture naming in chronic aphasia after TMS to part of right Broca's area: an open-protocol study. Brain Lang. 93, 95–105 10.1016/j.bandl.2004.08.004 15766771

[B56a] Naeser M. A. Martin P. I. Lundgren K. Klein R. Kaplan J. Treglia E. (2010). Improved language in a chronic nonfluent aphasia patient after treatment with CPAP and TMS. Cogn. Behav. Neurol. 23, 29–38 10.1016/j.bandl.2004.08.004 20299861 PMC2939495

[B56] Naeser M. A. Martin P. I. Theoret H. Kobayashi M. Fregni F. Nicholas M. (2011). TMS suppression of right pars triangularis, but not pars opercularis, improves naming in aphasia. Brain Lang. 119, 206–213 10.1016/j.bandl.2011.07.005 21864891 PMC3195843

[B57] Nitsche M. A. Paulus W. (2000). Excitability changes induced in the human motor cortex by weak transcranial direct current stimulation. J. Physiol. 527, 633–639 10.1111/j.1469-7793.2000.t01-1-00633.x 10990547 PMC2270099

[B58] Nitsche M. A. Paulus W. (2001). Sustained excitability elevations induced by transcranial DC motor cortex stimulation in humans. Neurology 57, 1899–1901 10.1212/WNL.57.10.1899 11723286

[B59] Oberman L. M. Pascual-Leone A. (2009). Report of seizure induced by continuous theta burst stimulation. Brain Stimul. 2, 246–247 10.1016/j.brs.2009.03.003 20160904 PMC2769021

[B60] Ohyama M. Senda M. Kitamura S. Ishii K. Mishina M. Terashi A. (1996). Role of the nondominant hemisphere and undamaged area during word repetition in poststroke aphasics - a PET activation study. Stroke 27, 897–903 10.1161/01.STR.27.5.897 8623110

[B61] Pascual-Leone A. Tormos J. M. Keenan J. Tarazona F. Canete C. Catala M. D. (1998). Study and modulation of human cortical excitability with transcranial magnetic stimulation. J. Clin. Neurophysiol. 15, 333–343 10.1097/00004691-199807000-00005 9736467

[B62] Pedersen P. M. Jorgensen H. S. Nakayama H. Raaschou H. O. Olsen T. S. (1995). Aphasia in acute stroke - incidence, determinants, and recovery. Ann. Neurol. 38, 659–666 10.1002/ana.410380416 7574464

[B63] Pierpaoli C. Jezzard P. Basser P. J. Barnett A. DiChiro G. (1996). Diffusion tensor MR imaging of the human brain. Radiology 201, 637–648 8939209 10.1148/radiology.201.3.8939209

[B64] Poreisz C. Boros K. Antal A. Paulus W. (2007). Safety aspects of transcranial direct current stimulation concerning healthy subjects and patients. Brain Res. Bull. 72, 208–214 10.1016/j.brainresbull.2007.01.004 17452283

[B65] Pulvermuller F. Hauk O. Zohsel K. Neininger B. Mohr B. (2005). Therapy-related reorganization of language in both hemispheres of patients with chronic aphasia. Neuroimage 28, 481–489 10.1016/j.neuroimage.2005.06.038 16099176

[B66] Robertson I. H. Fitzpatrick S. M. (2008). The future of cognitive neurorehabilitation, in Cognitive Neurorehabilitation, eds Stuss D. T. Winocur G. Robertson I. H. (New York, NY: Cambridge University Press), 565–574

[B67] Rosen H. J. Petersen S. E. Linenweber M. R. Snyder A. Z. White D. A. Chapman L. (2000). Neural correlates of recovery from aphasia after damage to left inferior frontal cortex. Neurology 55, 1883–1894 10.1212/WNL.55.12.1883 11134389

[B68] Rossi S. Hallett M. Rossini P. M. Pascual-Leone A. (2009). Safety, ethical considerations, and application guidelines for the use of transcranial magnetic stimulation in clinical practice and research. Clin. Neurophysiol. 120, 2008–2039 10.1016/j.clinph.2009.08.016 19833552 PMC3260536

[B69] Saur D. Lange R. Baumgaertner A. Schraknepper V. Willmes K. Rijntjes M. (2006). Dynamics of language reorganization after stroke. [Research Support, Non-U.S. Gov't]. Brain 129(pt 6), 1371–1384 10.1093/brain/awl090 16638796

[B70] Snodgrass J. G. Vanderwart M. (1980). Standardized set of 260 pictures - norms for name agreement, image agreement, familiarity, and visual complexity. J. Exp. Psychol. Hum. Learn. 6, 174–215 10.1037/0278-7393.6.2.174 7373248

[B71] Szaflarski J. P. Allendorfer J. B. Banks C. Vannest J. Holland S. K. (2013). Recovered vs. not-recovered from post-stroke aphasia: the contributions from the dominant and non-dominant hemispheres. Restor. Neurol. Neurosci. 31, 347–360 10.3233/RNN-120267 23482065 PMC3701454

[B72] Szaflarski J. P. Eaton K. Ball A. L. Banks C. Vannest J. Allendorfer J. B. (2011a). Poststroke aphasia recovery assessed with functional magnetic resonance imaging and a picture identification task. J. Stroke Cerebrovasc. Dis. 20, 336–345 10.1016/j.jstrokecerebrovasdis.2010.02.003 20719532 PMC2990790

[B73] Szaflarski J. P. Vannest J. Wu S. W. DiFrancesco M. W. Banks C. Gilbert D. L. (2011b). Excitatory repetitive transcranial magnetic stimulation induces improvements in chronic post-stroke aphasia. Med. Sci. Monit. 17, CR132–CR139 21358599 10.12659/MSM.881446 PMC3057942

[B74] Szelies B. Mielke R. Kessler J. Heiss W. D. (2001). Restitution of alpha-topography by piracetam in post-stroke aphasia. Int. J. Clin. Pharmacol. Ther. 39, 152–157 10.5414/CPP39152 11332870

[B75] Thiel A. Habedank B. Herholz K. Kessler J. Winhuisen L. Haupt W. F. (2006). From the left to the right: how the brain compensates progressive loss of language function. Brain Lang. 98, 57–65 10.1016/j.bandl.2006.01.007 16519926

[B76] Thiel A. Hartmann A. Rubi-Fessen I. Anglade C. Kracht L. Weiduschat N. (2013). Effects of noninvasive brain stimulation on language networks and recovery in early poststroke aphasia. Stroke 44, 2240–2246 10.1161/STROKEAHA.111.000574 23813984

[B77] Thulborn K. R. Carpenter P. A. Just M. A. (1999). Plasticity of language-related brain function during recovery from stroke. Stroke 30, 749–754 10.1161/01.STR.30.4.749 10187873

[B78] Tillema J. M. Byars A. W. Jacola L. M. Schapiro M. B. Schmithorst V. J. Szaflarski J. P. (2008). Cortical reorganization of language functioning following perinatal left MCA stroke. Brain Lang. 105, 99–111 10.1016/j.bandl.2007.07.127 17905426 PMC2763390

[B79] Turkeltaub P. E. Coslett H. B. Thomas A. L. Faseyitan O. Benson J. Norise C. (2012). The right hemisphere is not unitary in its role in aphasia recovery. Cortex 48, 1179–1186 10.1016/j.cortex.2011.06.010 21794852 PMC3221765

[B80] Turkeltaub P. E. Messing S. Norise C. Hamilton R. H. (2011). Are networks for residual language function and recovery consistent across aphasic patients? Neurology 76, 1726–1734 10.1212/WNL.0b013e31821a44c1 21576689 PMC3100133

[B81] Waldowski K. Seniow J. Lesniak M. Iwanski S. Czlonkowska A. (2012). Effect of low-frequency repetitive transcranial magnetic stimulation on naming abilities in early-stroke aphasic patients: a prospective, randomized, double-blind sham-controlled study. Scientific World Journal. 2012:518568 10.1100/2012/518568 23213288 PMC3508571

[B82] Warburton E. Price C. J. Swinburn K. Wise R. J. S. (1999). Mechanisms of recovery from aphasia: evidence from positron emission tomography studies. J. Neurol. Neurosurg. Psychiatry 66, 155–161 10.1136/jnnp.66.2.155 10071093 PMC1736204

[B83] Wasserman E. M. (1998). Risk and safety of repetitive transcranial magnetic stimulation: report and suggested guidelines from the International Workshop on the safety of repetitve transcranial magnetic stimulation, June 5-7, 1996. Electroencephalogr. Clin. Neurophysiol. 108, 1–16 10.1016/S0168-5597(97)00096-8 9474057

[B84] Weiduschat N. Thiel A. Rubi-Fessen I. Hartmann A. Kessler J. Merl P. (2011). Effects of repetitive transcranial magnetic stimulation in aphasic stroke a randomized controlled pilot study. Stroke 42, 409–415 10.1161/STROKEAHA.110.597864 21164121

[B85] Winhuisen L. Thiel A. Schumacher B. Kessler J. Rudolf J. Haupt W. F. (2005). Role of the contralateral inferior frontal gyrus in recovery of language function in poststroke aphasia - a combined repetitive transcranial magnetic stimulation and positron emission tomography study. Stroke 36, 1759–1763 10.1161/01.STR.0000174487.81126.ef 16020770

[B86] You D. S. Kim D. Y. Chun M. H. Jung S. E. Park S. J. (2011). Cathodal transcranial direct current stimulation of the right Wernicke's area improves comprehension in subacute stroke patients. Brain Lang. 119, 1–5 10.1016/j.bandl.2011.05.002 21641021

